# Kaempferol and zinc gluconate mitigate neurobehavioral deficits and oxidative stress induced by noise exposure in Wistar rats

**DOI:** 10.1371/journal.pone.0236251

**Published:** 2020-07-21

**Authors:** Isaac Oluwatobi Akefe, Joseph Olusegun Ayo, Victor Olusegun Sinkalu

**Affiliations:** 1 Department of Physiology, Biochemistry and Pharmacology, Faculty of Veterinary Medicine, University of Jos, Jos, Nigeria; 2 Department of Physiology, Faculty of Veterinary Medicine, Ahmadu Bello University, Zaria, Nigeria; University of Florida, UNITED STATES

## Abstract

This study investigated the effects of kaempferol and zinc gluconate on neurobehavioural and oxidative stress changes in Wistar rats exposed to noise. Thirty (30) rats were randomly divided into five groups: Groups I and II were administered with deionized water (DW); Group III, kaempferol (K); Group IV, zinc gluconate (Zn); Group V, kaempferol + zinc gluconate. Groups II, III, IV, and V were subjected to noise stress (N) induced by exposing rats to 100 dB (4 h/day) for 15 days, from day 33 to day 48 after starting the drug treatments. Neuromuscular coordination, motor coordination, motor strength, sensorimotor reflex, and learning and memory, were evaluated using standard laboratory methods. Levels of nitric oxide (NO), malondialdehyde (MDA) and activities of glutathione peroxidase (GPx), catalase and superoxide dismutase (SOD) were evaluated in the hippocampus. Exposure of rats to noise, induced significant neurobehavioural deficits and oxidative stress while the combined administration of kaempferol and zinc gluconate significantly (P < 0.05) improved open-field performance, motor coordination, motor strength, sensorimotor reflex, and learning and memory. Co-administration of kaempferol and zinc gluconate ameliorated noise-induced oxidative stress as demonstrated by the significantly increased activities of GPx, catalase, and SOD, and decreased levels of NO and MDA (P < 0.05 and P < 0.01 respectively), compared to the DW + N group. Our results suggest that oxidative stress, evidenced by increased NO and MDA concentration and decreased activities of GPx, catalase and SOD, were involved in the molecular mechanism underlying neurobehavioural impairment in Wistar rats, exposed to noise stress. Single treatment of kaempferol exerted a more potent mitigative effect than zinc gluconate, while their combination produced an improved outcome.

## Introduction

Recent years have witnessed a notable increase in the report of noise pollution and its attendant health impacts, especially in metropolitan environments [1–3]. Of particular interest is the adverse auditory effects, including temporary and/or irreversible hearing impairment [4], and non-auditory effects such as stress-related neurobehavioral deficits [5, 6], occurring due to excessive noise exposure. Interestingly, proteomics-based studies have uncovered several disrupted pathways attributed to noise blast exposures [7], and prominent among this is the alteration in ambient pressure within the ears alongside behavioural and motor deficits sequel to free radical-mediated oxidative stress [8]. Additionally, noise stress has been shown to increase the generation of reactive oxygen and nitrogen species which impair lipid and protein molecules, damage DNA, and trigger loss of neuronal function [9]. Importantly, psychoneurotic and psychosomatic complaints have been observed upon exposure to noise [10]. Similarly, Zheng and Ariizumi [11] reported that noise exposure prolongs healing of surgical wounds and also suppresses immune function [12]. Sustained exposure to sound levels greater than 85 dB, or sudden exposure to impulse noise lead to irreversible damage of sensory structures including the cochlea [13], and modifies the morphology of astrocytes [14]. Once damaged, the sensory hair cells in the cochlea do not regenerate, and ultimately results in permanent loss of hearing [2, 4]. Foregoing reports have shown that mice and rats exposed to high-noise intensity respond by freezing into a still stance, while exposure of rats to 95 dB at 0.5–5 kHz for a period of 5 minutes per day for 28 weeks, alters behavior and induces aggression. Likewise, in open-field behaviour, incessant noise of 85 dB amplifies defecation and decreases social and non-social activities, including sniffing, grooming or crawling of rats, compared with 50 dB, 65 dB or 75 dB [15].

The flavonoid, kaempferol belongs to the family *Zingiberaceae* [16] and appears as a yellow compound (3,5,7-trihydroxy-2-(4-hydroxyphenyl)-4H-1-benzopyran-4-one) with a molecular weight of 286.2 g/mol [17]. Abundant natural sources of kaempferol include green beans, spinach, green cabbage, amaranth, broccoli, horseradish, olive oil, tea, apple, tomatoes, chives, Chinese cabbage, cucumber, sweet potatoes, strawberries, cowpea, broccoli, grapes, aloe-vera, leek, onions, mustard, and lettuce [18]. Results of *in-vitro* and *in-vivo* studies suggest that kaempferol and its glycosides may possess beneficial effects in neurodegenerative diseases such as Alzheimer's and Parkinson's disease [19].

Zinc (Zn) is among the most abundant essential trace elements, second only to iron. More than 10% of all mammalian cellular proteins need Zn for their conformational transformation and/or activity [20–22] and it is required for many metabolic and enzymatic functions in the body [23]. Also, Zn performs vital neuromodulatory functions in the nervous system, including the inhibition of N-methyl-D-aspartate receptors and modulates glutamatergic excitation. Importantly, it prevents inhibition mediated by gamma-aminobutyric acid (GABA) by impeding the GABA-A receptor [24]. Zn is also an indispensable component for DNA-binding proteins with zinc fingers, copper/zinc superoxide dismutase (SOD) and a variety of proteins, involved in DNA repair [25]. Hence, zinc plays a vital role in transcription factor function, antioxidant defence, and DNA repair. Saki *et al*. [26] reported that zinc supplementation considerably improves the fertilization capacity of male rats, exposed to noise stress. Its supplementation in ethanol-intoxicated rats stabilizes increased activities of oxidative stress-related enzymes, catalase, superoxide dismutase, and glutathione peroxidase (GPx); suggesting its anti-peroxidative potential [27]. Documented reports show that zinc deficiency exacerbates the generation of ROS while its supplementation limits the formation of free radicals in rats [28]. Similarly, in young adults, increased levels of free radicals have been linked with impaired neurobehavioural performance, however, this impairment improves notably upon supplementation with zinc [4, 29]. As such, this study aimed to evaluate the mitigative effects of kaempferol and zinc gluconate on noise-induced neurobehavioral impairments and oxidative stress in Wistar rats.

## Methodology

### Animals

A total of 30 eight-week-old male Wistar rats, weighing between 110 and 120 g served as subjects. They were purchased from the breeding stock of the National Veterinary Research Institute, Nigeria, and maintained in plastic cages under standard laboratory conditions, in the animal holding facility. The rats were examined for health and pathogen-free status throughout the study and all efforts were made to minimize stress or discomfort to the animals during experimental procedures by ensuring minimal handling. The rats were given access to standard commercially-prepared rat pellet feeds (Vital Feeds, Jos, Nigeria) and water *ad libitum*. Animal welfare experts of the Ahmadu Bello University Committee on Animal Use and Care reviewed and approved all animal procedures (ABUCAUC No.: 02/17/22) in conformity with the National Institutes of Health guide for the care and use of laboratory animals (NIH Publications No. 8023, revised 1978).

### Study design

**Fig 1:**
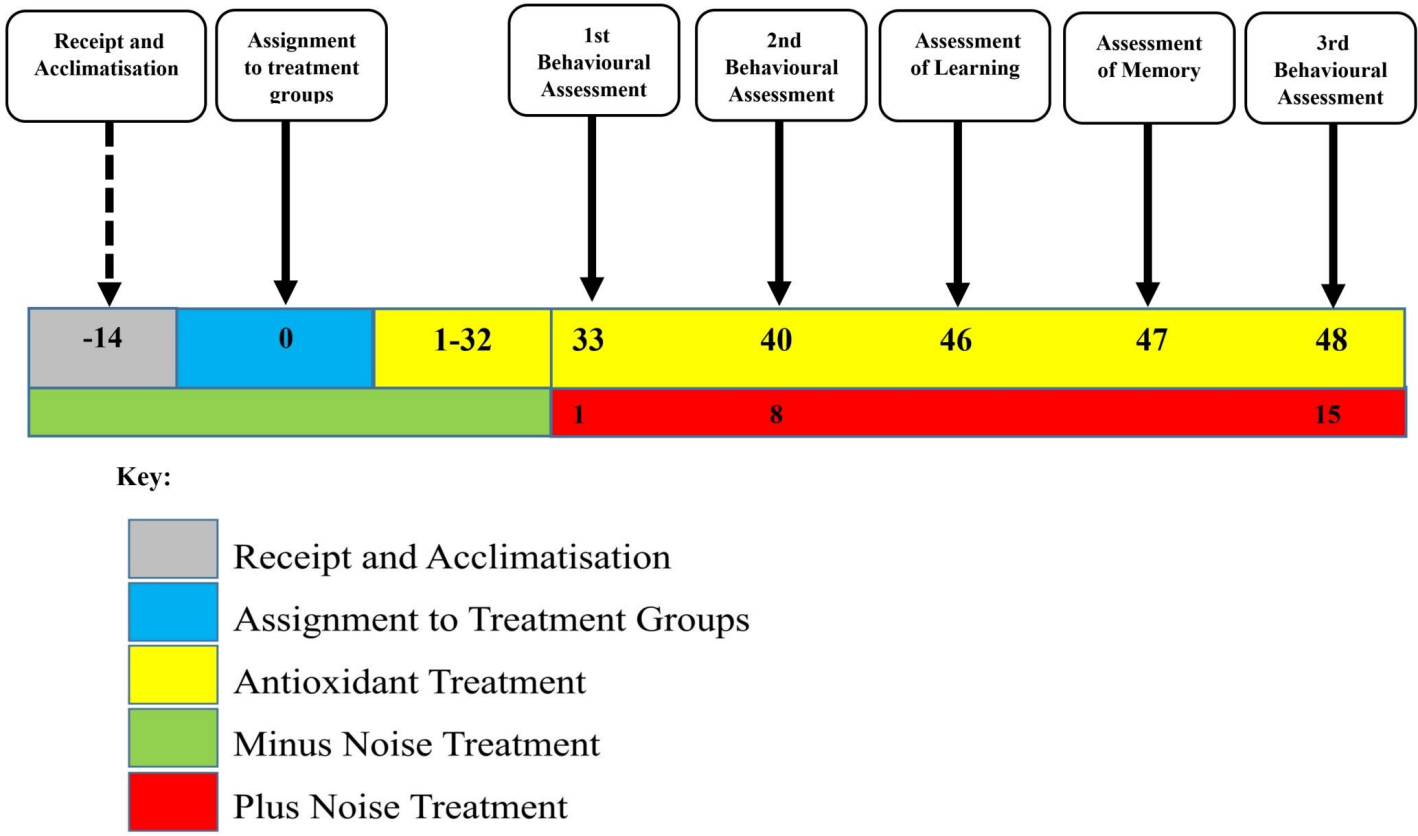
Experimental timeline.

Rats were divided into five groups by simple randomization, with each group consisting of six animals as follows: Groups I and II were administered with deionized water; Group III, kaempferol; Group IV, zinc gluconate; Group V, kaempferol + zinc gluconate for 34 days. Groups II, III, IV, and V were subjected to noise stress of 100 dB dose daily for four hours per day and for 15 days consecutively, from days 33–48 of the experimental period. Behavioral assessments were assessed in all groups on days 1, 8, and 15 after noise exposure; that is, on days 33, 40 and 48 of the experiment in all the rats (Fig 1). At the end of the experiment, the animals were anaesthetized by injection of ketamine + xylazine (16.25 mg/kg and 1.0 mg/kg respectively), i.p. and then rapidly decapitated.

### Drug and dosage

Kaempferol and zinc gluconate were purchased commercially (Aldrich Chemical Co. Ltd., Gillingham Dorset, England, Cat. No. 25,556–4) and administered orally at a dose of 5 mg/kg body weight [16] and 50 mg zinc/kg body weight [26, 30] respectively. They were reconstituted in deionised water (50 mg mL^-1^) prior to oral administration, while the controls were given deionised water only at a dose of 2 mL/kg body weight.

### Noise stress induction

Briefly, noise was produced by a loudspeaker (15 W), installed at a distance of 30 cm above the cages for Groups II-V, and driven by a white noise generator (DT-8850; Shenzhen Technology Co. Ltd., Shenzhen, Guangdong, China). The generator emitted frequencies in the range of 0–20 kHz and a precision sound level meter was used to set the intensity of sound to 100 dB uniformly in the cage. During the experiment, the noise level peaked at 100 dB/4 h/day for 15 days [5], with the noise intensity continuously measured using a digital sound level meter (Voltcraft SL-200, Hirschau, Germany). The intensity of 100 dB was chosen because it reflected the common noise level in industrial workplaces and noisy environments [31]. During noise exposure, the control group was kept in a different room with the uniform baseline noise level, and once the noise was switched off, they were returned to the same room with the other groups.

### Evaluation of neurobehavioural changes

To avoid bias, the neurobehavioral parameters were evaluated by trained observers blinded to the treatment schedule. The apparatus was cleaned between animals using 70% ethanol. All the rats were habituated in the test room for at least 30 min prior to evaluation of behavior which was carried out on days 1, 8, and 15 after the start of noise regiment as follows:

### Assessment of locomotion and emotional status (open field)

The evaluation of locomotion and emotional status was carried out on all experimental and control rats using the open-field apparatus as described by Zhu *et al*. [32]. The open-field apparatus was constructed using a cardboard box with a dimension of 50 × 50 × 45 cm with a clear Plexiglas on the inner surface (base). The floor of the box was divided into 25 equal squares. The parameters analyzed were locomotor activity and anxiety, which were assessed using horizontal locomotion (number of crossings of the lines marked on the floor) and the frequency of rearing. Assessing locomotor activity involved placing individual rats in the box and allowing it for a period of 3 minutes to walk freely and become familiarized with the environment. Thereafter and for the next 2 minutes, the number of squares crossed with all the paws by each rat was recorded. Rearing was measured by determining the frequency at which the rat stood on its hind-limbs; with the fore-paws lifted freely or leaned against the wall of the apparatus during the next 2 minutes, after the initial 3 minutes of habitation. The frequency of grooming (protracted washing of the coat), the number of feces and puddles of urine voided were observed and recorded using a camera.

### Assessment of neuromuscular coordination

The effect of the regimen on neuromuscular coordination was assessed using the inclined plane [33]. Each rat was placed on an apparatus made with an angled rough wooden plank, with a thick foam pad at its base. Angles were measured and marked on the apparatus beforehand and were obtained by propping the plank on a vertical bar with several notches. The plain was initially inclined at an angle of 35°, and, thereafter, increased step-wise by 5° until the rat could no longer hold on horizontally for 30 seconds without sliding down. The test was performed with each rat facing left and then right across the plank. The highest angle at which the rat could no longer stay, standing horizontally and facing any of the directions was recorded. Two trials were performed for each testing period.

### Assessment of motor balance

The motor balance was assessed using the beam-walk performance task as described by Abou-Donia *et al*. [33]. Briefly, the beam-walk performance was evaluated by allowing individual rat from each group to walk across a wooden black beam of 106-cm length, beginning at 17.2-cm width and ending at 1.0-cm width. Periodic widths were marked on the side of the apparatus. On each side of the narrowing beam, there was a 1.8-cm step-down to a 3.0-cm area, where each rat stepped, if necessary. As the rat walked across, the width of the beam at which it stepped down was recorded by one rater on each side, and this was repeated twice during each trial session.

### Assessment of motor strength

The fore-paw grip-time was evaluated using the method described by Abou-Donia *et al*. [33]. It was used to evaluate the motor strength of the rats hung from a 5-mm-thick diameter wood dowel, gripped with both fore-paws. The time spent by each rat before releasing its grip was recorded in seconds.

#### Assessment of sensorimotor reflex

The excitability score of each rat was evaluated using the method described by Ambali and Ayo [34]. Briefly, each rat was held by the tail upside down and kept in that position for 30 seconds. The response of each rat was rated using the ordinal scale of 0–5 as follows:

Grade 0: Rats did not show any sign of wriggling at all.
Grade 1: Rat wriggling was low with feeble fore-paw movement.
Grade 2: Rat responded through a stronger wriggling and forepaw movement.
Grade 3: Rat vigorously wriggled and showed strong fore-and hind-limb movement.
Grade 4: In addition to the observations in grade 3 above, the rat attempted unsuccessfully to climb on its tail.
Grade 5: In addition to the observations in grade 3 above, the rat successfully climbed the tip of its tail.

### Assessment of learning and short-term memory

The effect of the different regimens on learning was assessed using the step-down inhibitory avoidance task as described by Zhu *et al*. [32]. The effect was evaluated 48 hours prior to the termination of the experiment. It involved the use of an acrylic chamber 40 × 25 × 25 cm, consisting of a floor made of parallel 2-mm caliber stainless steel bars, spaced 1 cm apart. An electric current (80 volts) was delivered through the floor bars and a 2.5-cm high, 8 × 25 cm wooden platform, placed on the left extreme of the chamber. Each rat was gently placed on the platform. The ability of each rat to remain on the platform, having received an initial foot-shock when it stepped down from the platform the first time, was the basis of this assessment. The number of times the rat stepped down with all its limbs after receiving an electric shock was used as an index of learning. The maximum learning ability was observed when the rat stayed on the platform for 2 minutes without stepping down.

Short-term memory was assessed 24 hours later using the same procedure. In this case, each rat was again placed gently on the platform, and the period it remained on the platform without stepping down was recorded as an index of memory retention. The ceiling memory was observed when the rats remained on the platform for 2 minutes.

### Collection and analyses of biomarkers of oxidative stress

At the end of the experimental period, all the rats were sacrificed after anesthesia. Thereafter, brain tissues were collected and dissected to retrieve the hippocampus which was prepared as described previously by Shen et al. [35]. Briefly, brain tissue was immediately harvested and weighed over ice blocks. About 1mg of hippocampal tissue was removed and homogenized in 10mL of ice-cold phosphate-buffered solution (pH 7.0), centrifuged at 4^0^ C and 3000g for 5min. The supernatant was transferred into fresh Eppendorf tubes and stored at -20^0^ C till analysis to determine NO concentration, MDA concentration, and activities of GPx, SOD and catalase. Nitric oxide level was determined in brain homogenates using a commercially available kit (Biovision Inc., Milpitas, CA95035, U.S.A., Catalog #262–200) according to the manufacturer’s instructions. The concentration of MDA was determined using a commercially available colorimetric thiobarbituric acid reactive substances (TBARS) microplate assay kit, obtained from Oxford Biomedical Research, Oxford, MI48371, U.S.A. (Product number: FR40). The activity of GPx was measured using the GPx cellular activity assay kit (Biovision Inc., Milpitas, CA95035, U.S.A., Catalog #K762-100). Determination of the total SOD activity was carried out with the superoxide dismutase assay kit (Biovision Inc., Milpitas, CA95035, U.S.A., Catalog #462–100), according to manufacturer’s instructions. Catalase activity was determined using a commercially available kit (Biovision Inc., Milpitas, CA95035, U.S.A., Catalog #C290-100) according to the manufacturer’s instructions.

### Data analysis

Data obtained from the study were expressed as mean ± standard error of the mean (± SEM) and subjected to a repeated-measures analysis of variance (ANOVA), followed by Tukey’s *posthoc* test to determine if significant difference exists among the groups. Excitability scores were analyzed using the Kruskal-Wallis one-way analysis of variance on ranks followed by Dunn’s test. GraphPad Prism, version 6.0 for Windows (GraphPad Software, San Diego, CA, USA, www.GraphPad.com) was used to analyze all the data. Values of *P* < 0.05 were considered significant.

## Results

### Effect of kaempferol and zinc on open-field parameters in Wistar rats exposed to noise stress

#### Frequency of rearing

**Fig 2:**
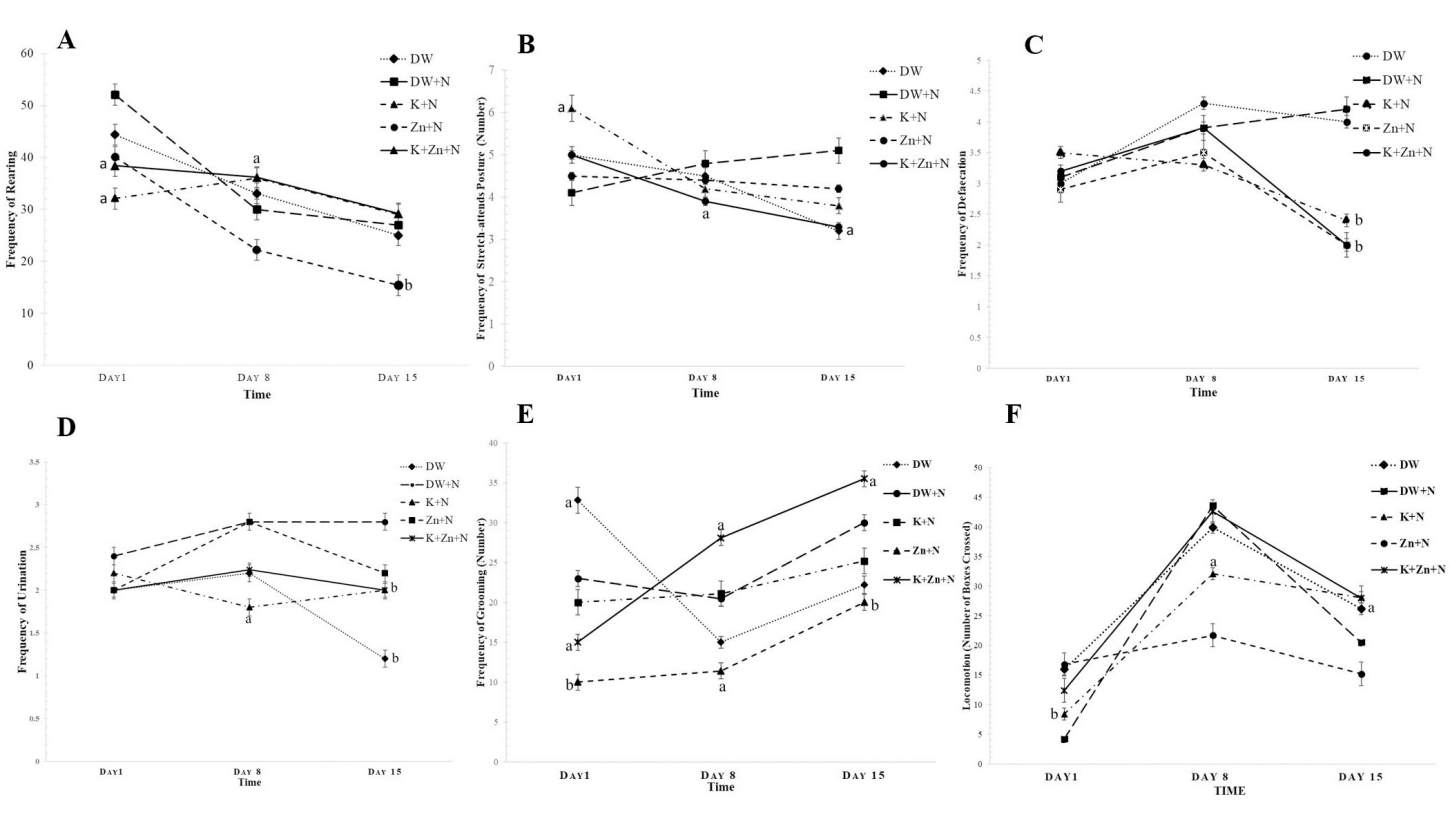
Effect of kaempferol and zinc on open-field parameters in Wistar rats exposed to noise stress (Mean ± SEM, n = 6). Individual rats were placed in the box and allowed to walk freely and become familiarized with the environment for a period of 3 minutes. Thereafter and for the next 2 minutes, the number of squares crossed with all the paws by each rat was recorded. Rearing was measured by determining the frequency at which the rat stood on its hind-limbs; with the fore-paws lifted freely or leaned against the wall of the apparatus during the next 2 minutes, after the initial 3 minutes of habitation. The frequency of grooming, the number of feces and puddles of urine voided were observed and recorded using a camera. A) Effect of kaempferol and zinc on the frequency of rearing. B) Effect of kaempferol and zinc on the frequency of stretch-attends postures (number). C) Effect of kaempferol and zinc on the frequency of defecation. D) Effect of kaempferol and zinc on the frequency of urination E) Effect of kaempferol and zinc on the frequency of grooming. F) Effect of kaempferol and zinc on locomotion. ^a^ *P* < 0.05, ^b^ *P* < 0.01, is significantly different compared to control group (DW+N). DW, Deionized water; K, Kaempferol; Zn, Zinc gluconate; N, Noise.

The frequency of rearing in groups treated with kaempferol + noise and kaempferol + zinc + noise increased relatively over the noise exposure period, compared to that of the group treated with deionized water + noise. The general pattern of the frequency of rearing across the treatment groups was that of a progressive decrease throughout the study period (Fig 2A). On day 1 of the noise period, the mean frequency of rearing obtained in the kaempferol + zinc + noise and kaempferol + noise treated groups were significantly lower (37.20 ± 1.44; *P* < 0.05 and 32.20 ± 1.00; *P* < 0.01) when compared to the deionized water + noise-treated rats (52.10 ± 0.60). However on day 8, the frequency in kaempferol + noise (37.90 ± 1.48) and kaempferol + zinc + noise (37.20 ± 1.44) groups were significantly higher (*P* < 0.05) than the value, obtained in deionized water + noise treated group (30.00 ± 0.42). On day 15, in the group treated with zinc + noise, the frequency (18.40 ± 2.64) was significantly (*P* < 0.01) lower than that recorded in the group treated with deionized water + noise (27.00 ± 2.21).

#### Stretch-attend postures

On day 1, the highest number of stretch-attend posture was observed in the group treated with kaempferol + noise (6.10 ± 1.40), which was significantly higher compared to that recorded in the deionized water + noise group (4.10 ± 2.00; *P* < 0.05). On days 8 and 15, the number of stretch–attend postures significantly decreased (*P* < 0.05) in the group treated with kaempferol + zinc + noise group (3.90 ± 0.48, 3.30 ± 3.10 respectively) when compared to the deionized water + noise group (4.80 ± 1.04, 5.10 ± 0.50, respectively). No significant difference was observed between the groups treated with kaempferol + noise, zinc + noise, and kaempferol + zinc + noise; Fig 2B).

#### Defecation

On day 15, the number of defecation in kaempferol + noise (2.49 ± 0.90), zinc + noise (2.0 ± 0.64) and kaempferol + zinc + noise (2.0 ± 0.00) groups, were significantly lower (*P* < 0.01), compared to the group treated with deionized water + noise (4.30 ± 2.24). No significant difference was observed between the kaempferol + zinc + noise treated group and the groups separately treated with kaempferol + noise and zinc + noise (Fig 2C).

#### Urination

On day 8, the frequency of urination significantly (*P* < 0.05) decreased in the kaempferol + noise group (1.80 ± 0.55), compared to the group treated with deionized water + noise (2.80 ± 0.82). On day 15, significant decrease in the urination frequency occurred in the groups treated with only deionized water (1.20 ± 0.50; *P* < 0.01), kaempferol + noise (2.00 ± 0.40; *P* < 0.05) and kaempferol + zinc + noise (2.0 ± 0.20; *P* < 0.05), compared to the deionized water and noise treated group (2.80 ± 0.00. There was no significant difference between the zinc + noise and deionised water + noise treated group; Fig 2D).

#### Grooming

As shown in Fig 2E, on day 1, the grooming pattern was significantly lower in the kaempferol + zinc + noise (15.00 ± 0.40) and zinc + noise (10.00 ± 0.80) groups (*P* < 0.05 and *P* < 0.01 respectively), compared to the control group, treated with deionized water + noise (23.00 ± 0.50). However, the deionised water treated group had significantly higher grooming (33.00 ± 0.50; *P* < 0.05) compared to the deionized water + noise treated group. On day 8, the grooming pattern recorded in kaempferol + zinc + noise-treated group was significantly higher (28.10 ± 0.80) than in those treated with deionized water + noise (20.48 ± 0.82; *P* < 0.05). The pattern of grooming in the zinc + noise treated group was significantly lower (11.40 ± 0.25; *P* < 0.05) than in the deionized water + noise treated group. On day 15, the grooming pattern in kaempferol + zinc + noise group was significantly higher (36.50 ± 0.70; *P* < 0.05) compared to the deionized water + noise group (30.0 ± 3.20).

#### Locomotor activity

On day 1 (Fig 2F), the locomotor activity in the groups treated with zinc + noise and kaempferol + zinc + noise (16.80 ± 4.00; *P* < 0.01 and 12.40 ± 0.20; *P* < 0.05 respectively) was significantly higher compared to the deionized water + noise group (4.20 ± 0.20). On day 8, the activity significantly decreased in the kaempferol + noise (32.10 ± 1.97; *P* < 0.05) and zinc + noise (21.70 ± 2.52; *P* < 0.01) groups compared to the deionized water + noise group (43.60 ± 1.47). On day 15, the groups treated with kaempferol + noise (28.10 ± 1.60) and kaempferol + zinc + noise (28.00 ± 1.40) showed a significantly higher (*P* < 0.05) activity, compared to that in the deionized water + noise (20.50 ± 0.80) group.

### Effect of kaempferol and zinc on neuromuscular coordination in Wistar rats exposed to noise stress

**Fig 3:**
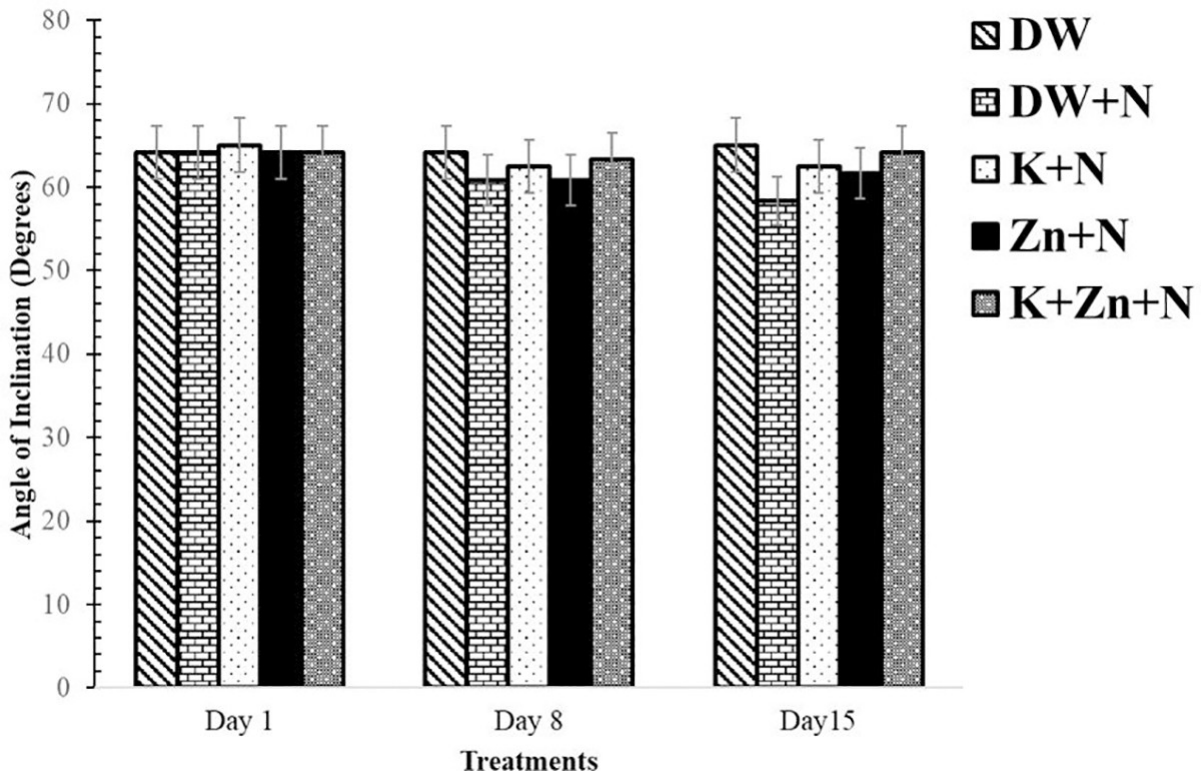
Effect of kaempferol and zinc on neuromuscular coordination in Wistar rats exposed to noise stress (Mean ± SEM, n = 6). Neuromuscular coordination was assessed using the inclined plane. The highest angle at which the rat could no longer stay, standing horizontally and facing any of the directions was recorded. Two trials were performed for each testing period. DW, Deionized water; K, Kaempferol; ZnG, Zinc gluconate; N, Noise.

There was relatively lesser coordination of movement in the deionized water + noise group (58.33 ± 2.11) on day 15, compared with values obtained in other groups, although the difference was insignificant. Additionally, on days 8 and 15, a higher coordination of movement was recorded in the animals in kaempferol + zinc + noise groups compared to the groups separately treated with kaempferol + noise and zinc + noise, however, the difference was not significant. Overall, on all the days, there was no significant difference in the neuromuscular performance of Wistar rats across the groups (Fig 3).

### Effect of kaempferol and zinc on motor balance in Wistar rats exposed to noise stress

**Fig 4:**
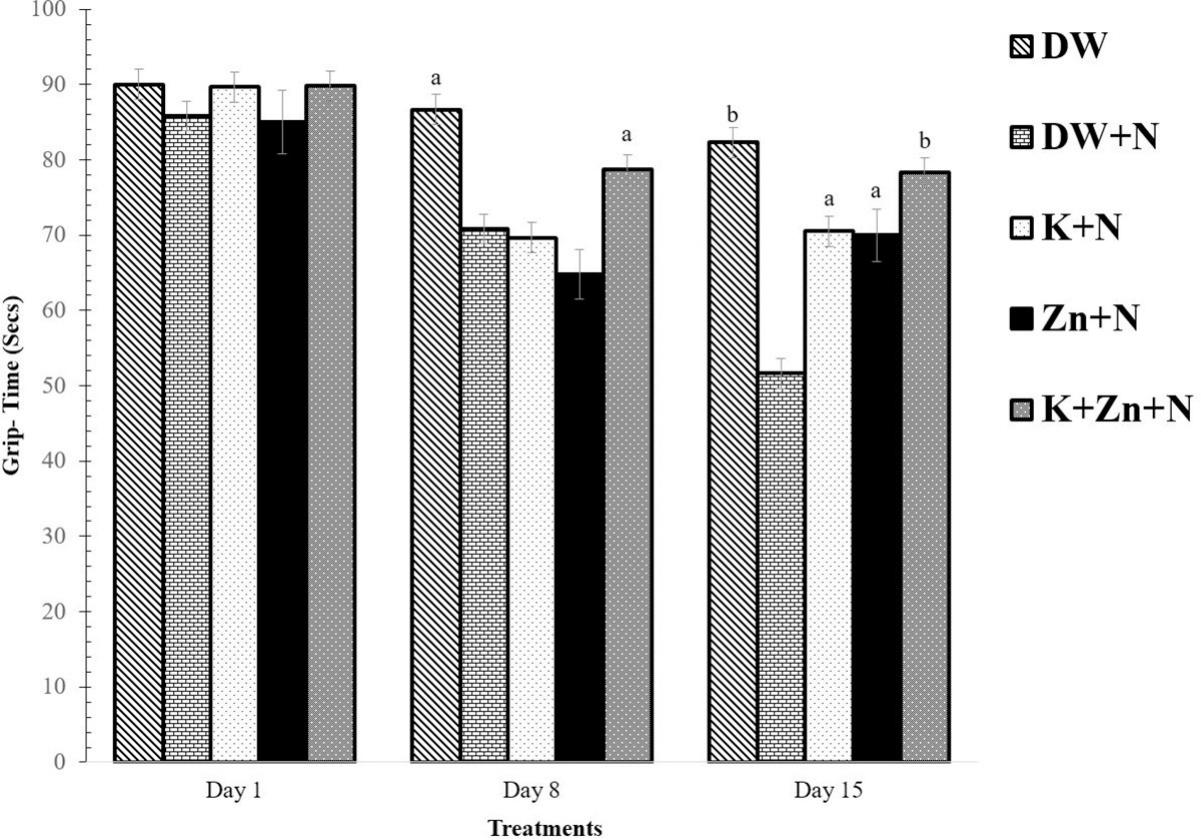
Effect of kaempferol and zinc on motor balance (beam walk) in Wistar rats exposed to noise stress (Mean ± SEM, n = 6). The motor balance was assessed using the beam-walk performance task. As the rat walked across, the width of the beam at which it stepped down was recorded by one rater on each side, and this was repeated twice during each trial session. ^a^ *P* < 0.05, ^b^ *P* < 0.01, ^c^ *P* < 0.001, is significantly different compared to control group (DW+N). * *P* < 0.05, is significantly different compared to K + Zn + N group. DW, Deionized water; K, Kaempferol; Zn, Zinc gluconate; N, Noise.

There was a significant difference (P < 0.05) in the step-down width of groups, exposed to noise stress and the unexposed group. The step-down width of the group treated with deionized water + noise was the highest on days 1, 8 and 15 (10.83 ± 1.40 cm, 11.9 ± 1.18 cm, and 19.50 ± 0.92 cm respectively); compared to the group treated with kaempferol + zinc + noise, which had the least width (5.33 ± 1.45 cm, 4.33 ± 1.41cm, 3.83 ± 1.35 cm) for the respective days. Additionally, the step-down width of the group treated with zinc + noise was significantly higher (P < 0.05) compared to the kaempferol + zinc + noise treated group. Also, on day 8, the motor balance in the kaempferol + zinc + noise group was significantly lower (P < 0.05) compared to the zinc + noise and kaempferol + noise treated groups. There was no significant difference (P > 0.05) between zinc + noise and kaempferol + noise treated groups on days 8 and 15 (Fig 4).

### Effect of kaempferol and zinc on motor strength in Wistar rats exposed to noise stress

**Fig 5:**
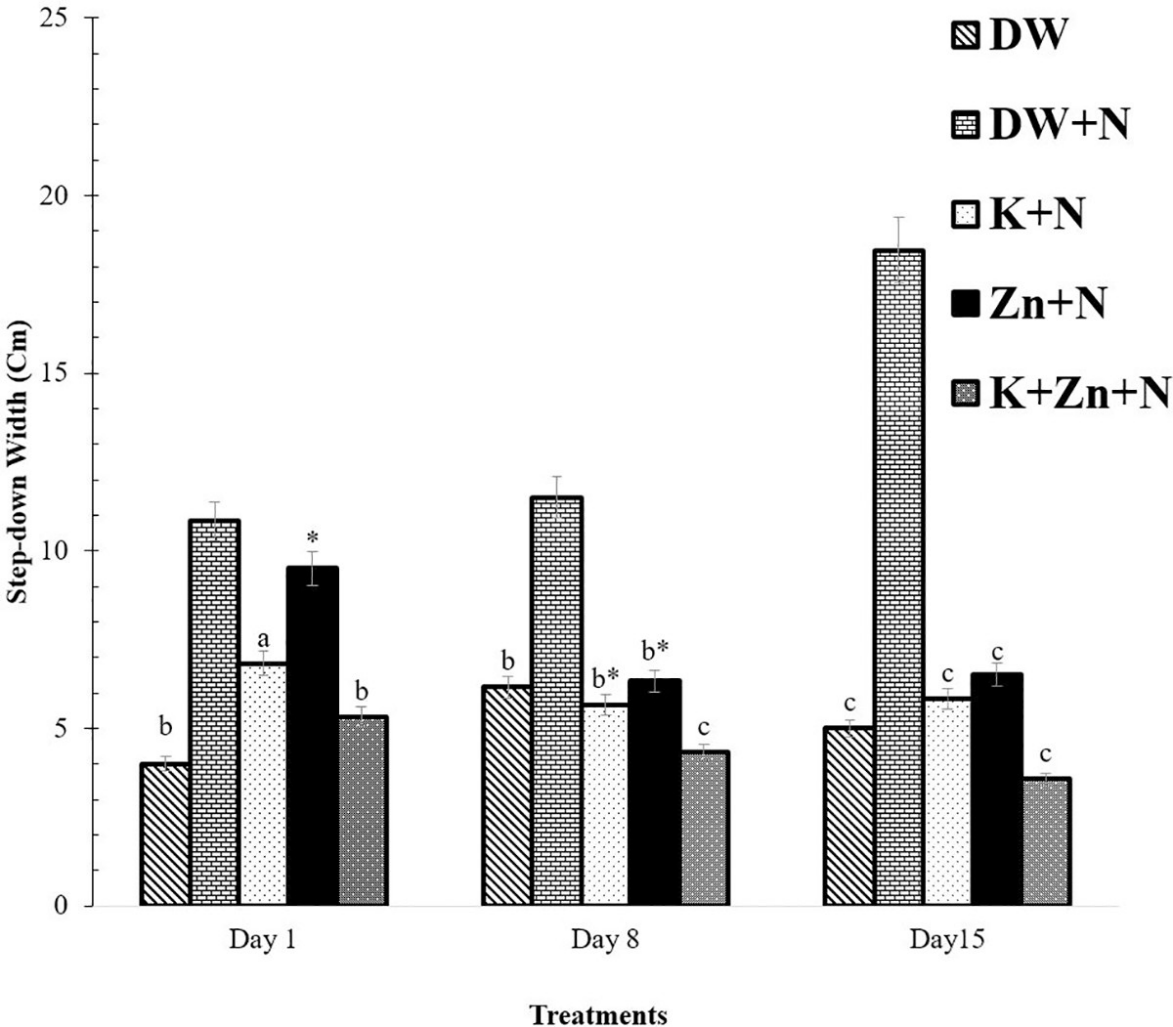
Effect of kaempferol and zinc on motor strength (fore-paw grip) in Wistar rats exposed to noise stress (Mean ± SEM, n = 6). The time spent by each rat before releasing its grip was recorded in seconds. ^a^ *P* < 0.05, ^b^ *P* < 0.01, is significantly different compared to control group (DW+N). DW, Deionized water; K, Kaempferol; Zn, Zinc gluconate; N, Noise.

The effect of treatments on motor strength is shown in Fig 5. On day 1, there was no significant (*P* < 0.05) difference in the time taken to hold onto a grip in rats belonging to the different groups. On day 8, there was a significant increase (*P* < 0.05) in the grip time of rats in the group treated with kaempferol + zinc + noise compared to the deionised water + noise treated group. There was however no significant difference between the groups treated with kaempferol + noise and zinc + noise and deionised water + noise. On day 15, the time taken to release the grip significantly increased in the group treated with kaempferol + zinc + noise (78.33 ± 2.69 s) compared to the groups treated with deionised water + noise (51.67 ± 2.29 s; *P* < 0.01), kaempferol + noise (71.11 ± 2.69 s; *P* < 0.05), and zinc + noise (70.00 ± 1.00 s; *P* < 0.05). Although, a lesser amount of time was spent holding the grip by animals separately treated with kaempferol + noise and zinc + noise compared to the kaempferol + zinc + noise group, however, the difference was not significant. Besides, there was no significant difference between the rats in groups treated with kaempferol + zinc + noise and deionised water only.

### Effect of kaempferol and zinc on sensorimotor reflex in Wistar rats exposed to noise stress

**Fig 6:**
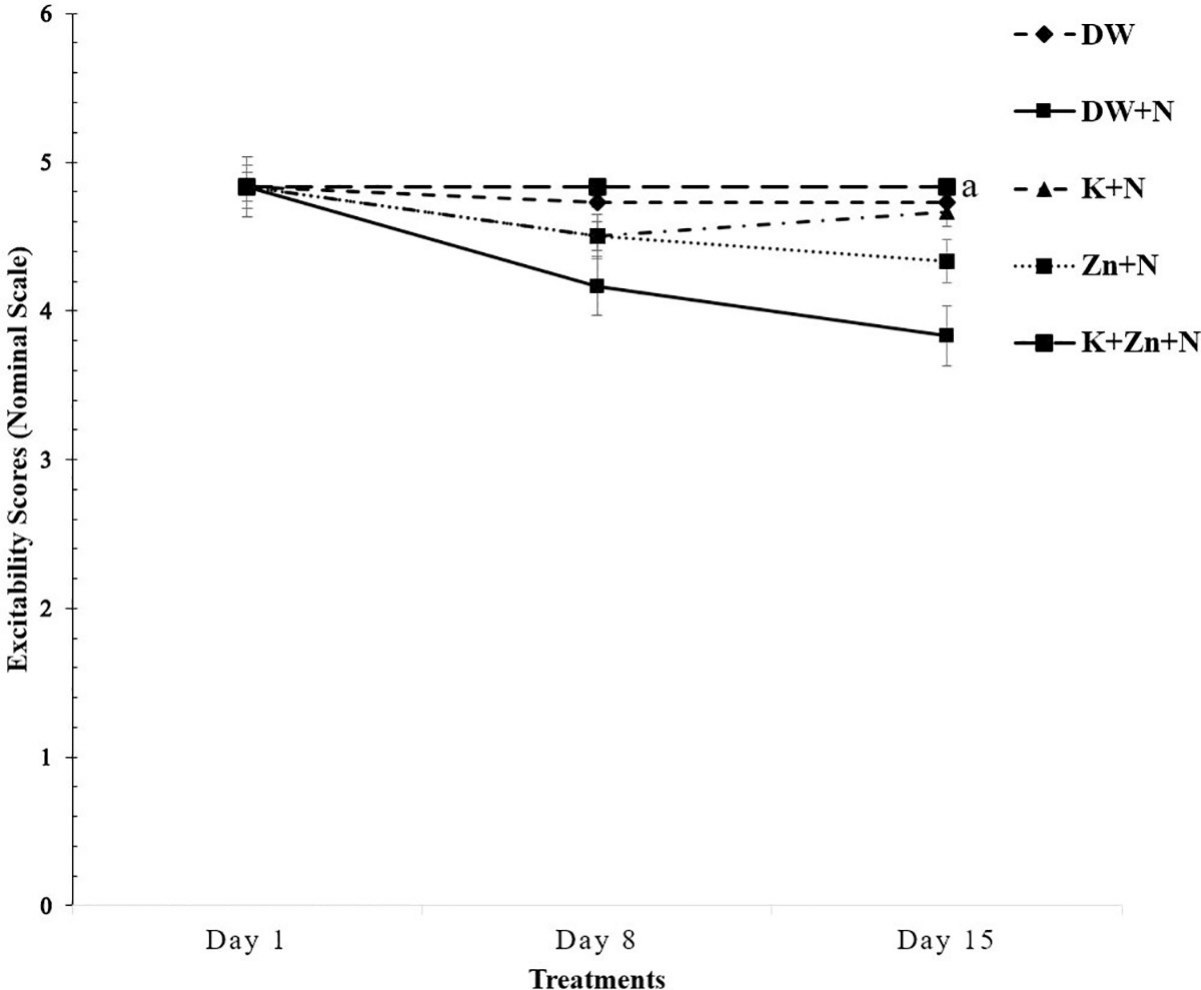
Effect of kaempferol and zinc on sensorimotor reflex in Wistar rats exposed to noise stress (Mean ± SEM, n = 6). ^a^ *P* < 0.05, is significantly different compared to control group (DW+N). DW, Deionized water; K, Kaempferol; Zn, Zinc gluconate; N, Noise.

There was no significant difference in the excitability scores of Wistar rats in all the treatment groups on days 1 and 8 of the noise exposure period (Fig 6). However, on day 15, the excitability score of kaempferol + zinc + noise (3.93 ± 0.01) treated group was significantly lower (P < 0.05) compared to that recorded in the DW + N group (4.98 ± 0.40). There was no significant difference between the Zn + N, K + N and DW + N treated groups. The highest sensorimotor reflex was reported in the DW + N group, while the lowest was seen in the K + Zn + N treated group.

### Effect of kaempferol and zinc on learning and memory in Wistar rats exposed to noise stress

**Fig 7:**
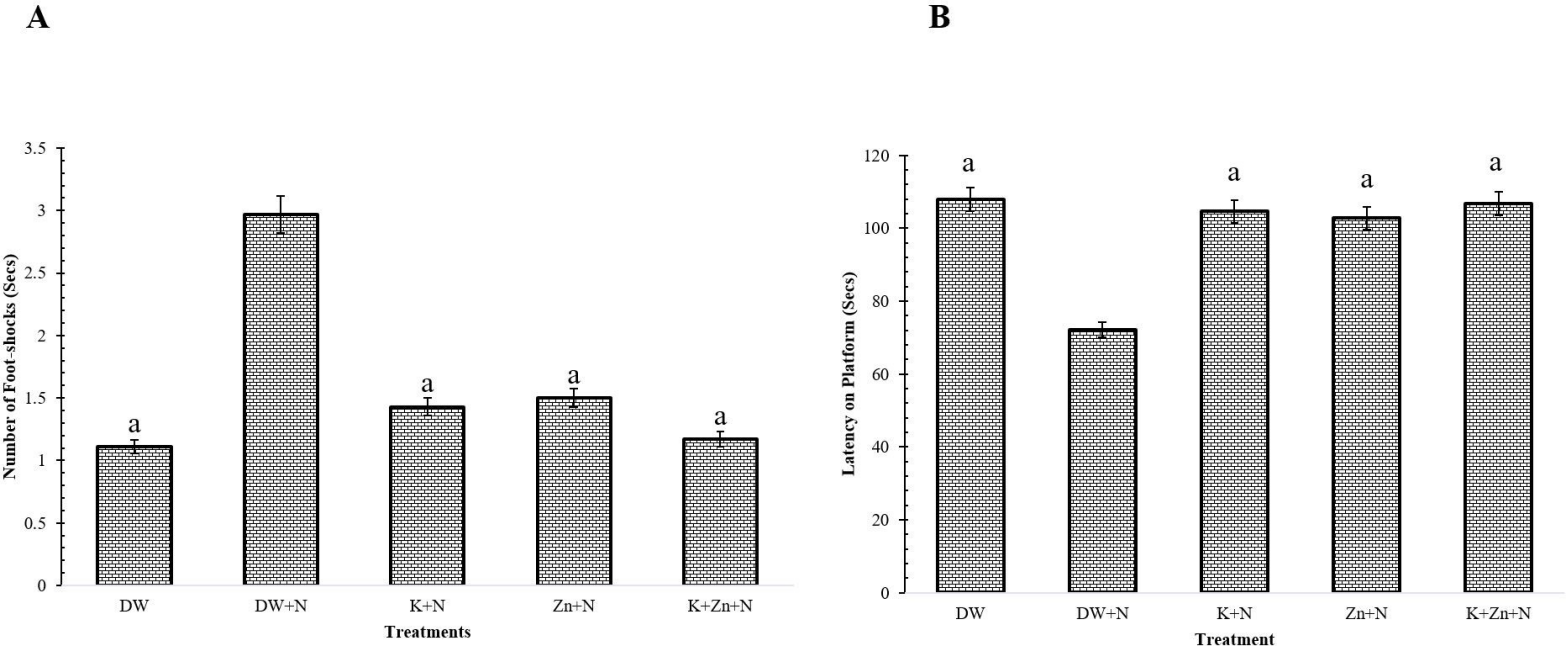
Effect of kaempferol and zinc on learning and memory in Wistar rats exposed to noise stress (Mean ± SEM, n = 6). A) Effect of kaempferol and zinc on learning. B) Effect of kaempferol and zinc on memory. Learning and memory assessment was carried out using the step-down inhibitory avoidance task and the number of times the rat stepped down with all its limbs after receiving an electric shock was used as an index of learning. The maximum learning and memory ability was observed when the rat stayed on the platform for 2 minutes without stepping down. ^a^ *P* < 0.05, ^b^ *P* < 0.01, is significantly different compared to control group (DW+N). DW, Deionized water; K, Kaempferol; Zn, Zinc gluconate; N, Noise.

The number of foot-shocks applied to the rats in the groups treated with kaempferol + zinc + noise (1.17 ± 0.17), kaempferol + noise (1.43 ± 0.21) and zinc + noise (1.50 ± 0.22) was significantly (*P* < 0.05) lower, compared to the deionized water + noise treated group. There was no significant difference (*P* < 0.05) in the number of foot-shocks applied to the rats in the kaempferol + noise and zinc + noise groups. Although, a higher amount of foot-shock was recorded in the animals separately treated with kaempferol + noise and zinc + noise compared to the kaempferol + zinc + noise and deionised water-treated groups, however, the difference was not significant. The highest number of foot-shocks was recorded in the deionized water + noise treated group (2.97 ± 0.31), while the least number was reported in the kaempferol + zinc + noise group (Fig 7A).

A significant (P < 0.05) difference was observed in memory between the groups. The amount of time (seconds) spent on the platform by the group treated with only deionised water (108 ± 2.08 s), and kaempferol + zinc + noise (106.80 ± 2.14 s) was significantly higher, compared to those recorded in the groups treated with deionized water + noise (72.21 ± 2.11 s; *P* < 0.01), kaempferol + noise (104.70 ± 1.80 s; *P* < 0.05) and zinc + noise (102.80 ± 0.14 s; *P* < 0.05). Additionally, no significant difference was recorded in the groups separately treated with kaempferol + noise and zinc + noise, but they were however significantly higher (*P* < 0.05), compared to that recorded in the deionized water + noise group (Fig 7B). Although, a lesser amount of time was spent on the platform by animals separately treated with kaempferol + noise and zinc + noise compared to the kaempferol + zinc + noise group, however, the difference was not significant.

### Effect of kaempferol and zinc gluconate on oxidative stress in Wistar rats exposed to noise stress

There was a significant difference (*P* < 0.05) in NO levels (nmol/μL) evaluated in hippocampal section of brain homogenates of rats in the K + Zn + N treated group (1.29 ± 0.13) which was lower than that in the DW + N group (1.95 ± 0.18). However, the nitric oxide levels in the groups separately treated with Zn + N and K + N did not vary significantly (*P* > 0.05) compared with each other and to the K + Zn + N group. There was no significant difference (*P* > 0.05) between the groups treated with DW only and K + Zn + N. The lowest NO level was reported in the K + Zn + N group, while the highest concentration was seen in the DW + N group (Table 1).

**Table 1:** Effect of kaempferol and zinc gluconate on concentrations of brain nitric oxide and malondialdehyde, and activities of glutathione peroxidase, catalase and Superoxide dismutase in Wistar rats exposed to noise stress (Mean ± SEM, n = 6).

				Groups		
		DW	DW + N	Zn + N	K + N	K + Zn + N
**Parameters**						
	Nitric oxide (nmol/μL)	1.33 ± 0.14^a^	1.95 ± 0.18	1.53 ± 0.10	1.50 ± 0.14	1.29 ± 0.13^a^
	Malondialdehyde (μΜ)	3.05 ± 0.51^a^	5.99 ± 0.13	3.13 ± 0.10^a^*	3.01 ± 0.21^a^	2.52 ± 0. 15^b^
	Glutathione peroxidase (nmol/mg protein)	6.72 ± 0.02^b^	4.21 ± 0.14	5.62 ± 0.33^a^*	5.65 ± 0.97^a^*	6.70 ± 0.01^b^
	Superoxide dismutase (u/mg protein)	6.80 ± 0.01^b^	4.15 ± 0.11	5.22 ± 0.21^a^*	5.26 ± 0.22^a^*	6.99 ± 0.15^b^
	Catalase (IU/L)	41.90 ± 0.14^b^	25.70± 1.74	33.25 ± 0.88^a^	34.65 ± 1.14^a^	38.2 ± 1.22^b^

On day 15 following the beginning of noise exposure, the animals were sacrificed and brain tissue collected. About 1mg of hippocampal tissue was removed and homogenized in 10mL of ice-cold phosphate-buffered solution (pH 7.0), centrifuged at 4^0^ C and 3000g for 5min. The supernatant was retrieved and used to determine NO concentration, MDA concentration, and activities of GPx, SOD and catalase. ^a^ *P* < 0.05, ^b^ *P* < 0.01, is significantly different compared to control group (DW+N). * *P* < 0.05, is significantly different compared to Zn + K + N. DW, Deionised water; K, Kaempferol; Zn, Zinc gluconate; N, Noise.

Malondialdehyde values (μΜ) in brain homogenates of rats were significantly lower in the K + Zn + N (*P* < 0.01) as well as in Zn + N, K + N and DW groups (*P* < 0.05) compared to the DW + N group. Although the values of MDA in the Zn + N was significantly higher (*P* < 0.05) when compared with the K + Zn + N group, there was no significant variation between Zn + N, K + N and DW groups. The lowest MDA level was reported in the K + Zn + N group, while the highest concentration was seen in the DW + N group (Table 1).

GPx activity was measured (nmol/mg protein). The activities of GPx in both the DW (6.72 ± 0.02) and K + Zn + N (6.70 ± 0.01) groups were significantly higher (*P* < 0.01) compared with the DW + N (4.21 ± 0.14). Although there was no significant variation between the groups separately treated with Zn + N and K + N, they were however significantly higher (*P* < 0.05) when compared with the DW + N group and significantly lower (*P* < 0.05) compared with the K + Zn + N group. The highest GPx activity was reported in the DW group, while the lowest was seen in the DW + N treated group (Table 1).

The SOD activity was measured (u/mg protein). The activities of SOD in both the DW and K + Zn + N groups were significantly higher (*P* < 0.01) when compared with the DW + N. Although there was no significant variation in activities of SOD between the groups separately treated with Zn + N and K + N, they were however significantly higher (*P* < 0.05) when compared with the DW + N group and significantly lower (*P* < 0.05) compared with the K + Zn + N group. The highest SOD activity was reported in the DW group, while the lowest was seen in the DW + N treated group (Table 1).

Catalase activity was measured (IU/L) and the activities in both DW and K + Zn + N groups were significantly higher (*P* < 0.01) when compared with the DW + N. Similarly, although there was no significant variation between the groups separately treated with Zn + N and K + N, they were however significantly higher (*P* < 0.05) when compared with the DW + N group. The highest catalase activity was reported in the DW group, while the lowest was seen in the DW + N treated group (Table 1).

## Discussion

### Effect of treatments on open-field parameters in Wistar rats exposed to noise stress

In the absence of locomotor alterations, increased exploration as indicated by increased rearing is interpreted as anxiolytic effects, whereas decreased exploration as an anxiogenic effect. Thus, exposure of rats to noise in the present study resulted in anxiogenic effects. The mechanism of the anxiogenic effect of noise stress may involve increased activity of the hypothalamo-pituitary-adrenal axis, and alterations in dopaminergic and serotonergic neurotransmissions in the hypothalamus [5]. Similarly, results from our study showed that administration of zinc significantly reduced the frequency of rearing, suggesting a potential anxiogenic effect. Previous studies have shown that aside from the influence of zinc on the process of glutamate neurotransmission, other complex theories about the influence of zinc on GABA-ergic neurotransmission has been reported. Molecular studies have shown that zinc has bidirectional modulatory effects on particular GABA receptors, which are mostly characterized in the hippocampus. In this way, zinc is possibly included in the process of GABA-ergic neuron plasticity, depending on the neurons’ sensitivity to the zinc effect and also depending on the influence of glutamate neurotransmission [36]. This bi-directional effect of separately administering zinc alone may be responsible for the results observed on some days in this study. The insignificant difference observed between the K + N and K + Zn + N groups suggest that the highest frequency of rearing observed on day 8, in the kaempferol and zinc gluconate treated group, was primarily due to the mitigating potential of kaempferol to mitigate the anxiogenic effect induced by noise [37] rather than an additive effect of zinc gluconate. In the present study, the exposure of Wistar rats to noise increased stretch-attends postures, demonstrating an anxiogenic effect. The result of the stretch-attends postures further buttressed the finding that noise produces an anxiogenic effect [38], found to be ameliorated in the present study by treatment with kaempferol and zinc gluconate.

Previous literature have reported that exposure to noise increases the frequency of defecation, however, on day 15 of our study, there was no significant difference between the frequency of defecation in the DW + N group and the DW group. This may be due to a progressive habituation of the animals to continuous noise exposure, and consequently administration of kaempferol and/or zinc produced a significant anxiolytic effect compared to the DW + N group and the DW group. Also, the higher frequency of urination recorded in the open-field assessment of deionised water + noise-exposed rats, compared with that obtained in the groups treated with kaempferol and zinc gluconate separately and/or in combination was an evidence of increased anxiety. This finding agreed with the report of Brown *et al*. [37] who reported that noise increased anxiety in exposed animals. It also suggests that the effect of combining kaempferol and zinc gluconate was not additive, however, kaempferol was more potent than zinc in mitigating the anxiogenic effect of noise.

The significant increase in locomotion and grooming observed in the noise stress-exposed groups treated with kaempferol and zinc, compared to the group treated with deionized water + noise, corroborated the finding of Wankhar *et al*. [5]; who demonstrated that the antioxidant effect of vitamin C content in *E*. *officinalis*, improved locomotor activity in Wistar rats, exposed to noise stress. They also showed that stress induced an increase in corticotrophin-releasing hormone and corticosterone, which is responsible for the decreased locomotor activity, recorded in the present study. Therefore, the antioxidants, kaempferol and zinc gluconate may reduce secretion of corticotropin-releasing hormone into the bloodstream, inducing significant behavioural protection in the rats. The mechanism by which kaempferol mitigated the alteration in the behavioural parameters may be due to its anti-oxidative potential. Kaempferol exerts antioxidant effects by inhibiting the activities of enzymes that generate ROS, such as xanthine oxidase [39]. Like other flavonoids, kaempferol may also enhance the formation of hydroxyl radical through the Fenton’s reaction by chelating ferrous or cuprous ions [38, 40]. It may also exert antioxidant effects by increasing the expression or activity of antioxidant enzymes, such as GPx and haeme oxygenase-1 [41–42]. Kaempferol and its glycosides also prevent lipid peroxidation of cytomembranes [43]; with the activity of kaempferol being higher than that of its glycosides owing to its higher lipophilic capacity to penetrate lipid bilayers to exert its activities.

### Effect of treatments on neuromuscular coordination in Wistar rats exposed to noise stress

The finding that the neuromuscular performance showed no significant effect demonstrated that noise stress did not impair the coordination of neuromuscular activity of rats; therefore, the pre-treatment of noise stress-exposed rats with kaempferol and zinc gluconate did not exert beneficial effects. This result contradicts the report of Tao *et al*., [44] who reported that noise stress induces impairment in neuromuscular coordination. The difference in these results may be due to the variation in the age of the subjects used for the study as well as variation in the study environment.

### Effect of treatments on motor balance in Wistar rats exposed to noise stress

Beam walking across bridges of different cross-sections provides a well-established method of monitoring motor coordination and balance in rats. The result of the present study showed a significant decrease in the step-down width of the groups exposed to noise and treated with zinc and kaempferol separately; and in the group given their combination, compared to the group treated with deionized water + noise. The increase in latency and number of foot slips in the noise-exposed group treated with deionized water showed a progressive increase in motor impairment. This study, therefore, revealed that noise stress caused muscular incoordination, which was evident in rats treated with deionized water + noise only. The cerebellum plays a role in maintaining posture through a servocomparator function, which may be impaired in rats exposed to noise stress [5, 6]. The distorting signals from the auditory labyrinth and proprioceptive signals from different parts of the body may have abolished the corrective signals involved in the adjustment of muscle tone, leading to impairment of posture and balance. The cerebellum plays a major role in maintaining equilibrium during rapid motions, by rapidly changing the direction through a predictive function. The basal ganglia may also be implicated in noise stress as it is involved in the control of the muscle tone and voluntary movements, while the caudate nucleus stimulates muscle tone through stimulation of vestibular nucleus and inferior olive. The ganglia receive projection fibers from the motor cortex to *corpus striatum*, *globus pallidus* and several nuclei in the brainstem; and the neurons may be implicated in the interruption of the nerve pathway, controlling muscle tone [39]. The result of the present study showed that the administration of kaempferol and zinc gluconate considerably ameliorated the impairment in motor coordination which may have decreased the foot-slip width, and thus, accentuating the role of oxidative stress in brain motor dysfunction [44].

### Effect of treatments on motor strength in Wistar rats exposed to noise stress

The fore-paw grip test, used to assess the motor strength and integrity of the muscle showed that on day 8, the groups separately treated with kaempferol and zinc showed less grip-strength compared to the deionised water + noise treated group. Whilst there is a trend to a decrease, this did not reach a significant level (P > 0.05). The observation that at day 8 there were decreases in all the treatment groups but by day 15 the noise treated rats continued to decrease whilst the antioxidant treatments prevented this, suggests that the exposure to noise had a continual and accumulative detrimental effect on the rat’s grip-strength whilst the antioxidant treatments moderated the severity of these deficits. This finding corroborated the result of Wankhar *et al*. [5], who reported that noise stress is a potent stressor, capable of disrupting physical, emotional and mental homeostatic behaviors. The mechanism underlying the homeostatic impairments may be due to increased generation of ROS, and consequently, decrease in the activities of the antioxidant enzymes; SOD, catalase and GPx resulting in increased lipid peroxidation of cytomembranes, neuronal degeneration and DNA damage [44, 45]. The mitigative effects of these antioxidants in the present study were demonstrated by the increased fore-paw grip-time recorded in the groups treated with kaempferol and/ zinc gluconate on day 15, after noise exposure.

### Effect of treatments on sensorimotor reflex in Wistar rats exposed to noise stress

There was a significant decrease in the excitability score of the group treated with kaempferol + zinc + noise compared to that of the group treated with deionized water + noise only. The progressive decrease in the excitability scores of rats treated with kaempferol and zinc reflects the state of improved memory and mental alertness. The increased excitability scores recorded in the group treated with deionized water + noise is indicative of a deficit in memory which may be due to increased concentration of NO, impairment in the activities of SOD, catalase and GPx and consequently, in movement-related neuronal activities. The present finding corroborates the report of Haberman *et al*. [46], who reported heightened cortical excitability in aged rats with memory impairment. Treatment with kaempferol and zinc gluconate significantly improved the excitability scores, plausibly due to enhancement in the activities of brain GPx, catalase and SOD enzymes [4, 27, 47].

### Effect of treatments on learning and memory in Wistar rats exposed to noise stress

The result of the present study showed that noise induces impairment in learning and memory by causing increased oxidative stress. The finding agrees with that of Tao *et al*. [44], who showed that impairment in learning and memory increase due to oxidative stress-induced neuronal degeneration, and suppression of neurogenesis of auditory nuclei in Wistar rats, exposed to noise stress. The significant increase in memory observed in the group treated with the combination of kaempferol and zinc, compared to the groups separately treated with kaempferol and zinc, indicates that kaempferol and zinc synergistically ameliorate impairment in memory of Wistar rats, exposed to noise stress. The result corroborates previous reports on the role of flavonoids in mitigating noise-induced memory and cognition impairments [44, 45]. Thus, kaempferol and zinc gluconate, which are potent antioxidants, exert mitigating effects on noise-induced impairment in the learning and memory of Wistar rats.

### Effect of treatments on malondialdehyde concentration in Wistar rats exposed to noise stress

Studies have shown that catalase, SOD and GPx are among the most important endogenous antioxidative enzymes with activities indicative of oxidative stress. Likewise, in assessing the level of oxidative damage, nitric oxide and malondialdehyde concentrations have been reported to be suitable.

The increased MDA concentration recorded in this study demonstrated that noise stress increased ROS-induced cell damage and death [8, 9]; which agreed with the findings that noise stress increases lipid peroxidation, evidenced by increased MDA concentration [48]. The result of the present study showed, for the first time, that co-administration of kaempferol and zinc gluconate mitigated noise stress-induced increase in MDA concentration in Wistar rats. The significant decrease in MDA concentration observed in kaempferol and zinc gluconate-treated rats, compared to rats separately treated with kaempferol and zinc gluconate, indicates that kaempferol and zinc gluconate acted additively to ameliorate adverse effects of noise stress, induced via increased lipid peroxidation in Wistar rats. The finding agreed with the result obtained by Shrivastava *et al*. [46], who reported that kaempferol decreases MDA concentration in Wistar rats, exposed to acrylamide intoxication. One of the major oxidation products of peroxidized polyunsaturated fatty acids is MDA [39], and an increase in MDA concentration is indicative of oxidative stress and lipid peroxidation. Ultimately, lipid peroxidation leads to oxidative degradation of polyunsaturated fatty acids and its occurrence in biological membranes impair membrane fluidity and activation of several membrane bound-enzymes that are crucial for many biological processes [49]. This is suggested to be liable for the impairment in neurobehavior observed in this study. The result of the present study suggests that kaempferol prevented noise-induced lipid peroxidation which agreed with the finding of Hou *et al*. [43] who reported that kaempferol and its glycosides inhibit free-radical-initiated lipid peroxidation of human erythrocyte ghost cells. The result of the present study also corroborates that of Nunes *et al*. [27] and Khalaf *et al*. [50] who reported that zinc supplementation in ethanol-intoxicated rats considerably increase activities of antioxidant enzymes and stabilize MDA concentration, thus signifying its anti-peroxidative potential.

### Effect of treatments on Nitric Oxide (NO), GPx, SOD and catalase activities in Wistar rats exposed to noise stress

The result of this study demonstrated that kaempferol and zinc gluconate decreased the levels of brain NO in the antioxidant-treated groups, compared to the DW+N group which showed a significant increase in the levels of NO. The increase in NO level may be due to elevated NO synthase activity and elevated nitrate/nitrite levels in the brain [51]. Increased generation of ROS induced by elevated corticosterone levels in response to noise stress can also explain increased NO concentration via enhancing the inactivation of NO [52]. Also, free radicals may induce signal transduction pathways for nitric oxide synthase (iNOS) [53]. NO is an oxygen-free radical with harmful effects on the nervous system and the increased levels observed in the DW + N group is congruent with the impaired neurobehaviour observed. This finding is in agreement with the study of Said and El-Gohary who stated that exposure to noise stress increased the levels of nitric oxide in adult male albino rats [54]. The decreased NO levels observed the antioxidant treated group suggests that kaempferol and zinc gluconate ameliorated noise stress by scavenging free radicals responsible for elevated brain NO levels.

Importantly, our result showed that kaempferol and zinc gluconate increased the activity of brain catalase, GPx, and SOD in the antioxidant-treated groups, compared to the DW+N group. This increase was significant in the groups treated with kaempferol and zinc gluconate, indicating that kaempferol and zinc gluconate ameliorate noise stress-induced oxidative stress in Wistar rats. This finding agrees with the result obtained by Shrivastava *et al*. [55], who reported that kaempferol restores acrylamide intoxication-induced decrease in GPx, activities in Wistar rats. Kaempferol has also been demonstrated to exert its antioxidant effects by increasing the activity of antioxidant enzymes; catalase, SOD, GPx, and haeme oxygenase-1, and by enhancing the antioxidant status of animals via improvement of the free radical-scavenging potential of the enzymes [56]. This result is also in harmony with previous reports that zinc plays a principal function in the maintenance of the integrity of Cu-Zinc SOD as a co-factor and also in regulating glutathione, which is crucial in cellular antioxidant defence mechanisms [57]. Yeh et al [4] also reported the mitigating effect of oral zinc supplementation on patients with noise-induced hearing loss associated tinnitus. Furthermore, zinc stabilises the thiol pool by protecting the sulfhydryl group, hence it shields from oxidation [58]. The collective effect of zinc and its role as a co-factor for many enzymes involved in metabolic processes may contribute to the mitigation of noise stress in rats. The results obtained from the current study corroborate previous findings in our laboratory, which showed that treatment with kaempferol and zinc, singly and in combination ameliorated noise-induced changes in erythrocyte osmotic fragility and haematological parameters of Wistar rats [59]. It is therefore suggested that kaempferol and zinc gluconate may improve oxidative stress by enhancing the activation of antioxidant proteins, molecules, and enzymes.

Further studies of specific regions of the brain and other behavioural test models are required to determine the molecular mechanism of noise stress. Besides, the optimum dose of kaempferol and zinc gluconate required for the mitigation of noise stress should be ascertained. The result of the present study suggests that high-noise risk population may benefit from increased consumption of foods, containing the antioxidants, kaempferol and zinc.

## Conclusion

Our data demonstrate that exposure of Wistar rats to noise induced oxidative stress and lipid peroxidation, which may be implicated in the molecular mechanism underlying the observed changes in neurobehaviour. Single and combined treatment with kaempferol and zinc gluconate ameliorated noise-induced alterations in oxidative status and neurobehaviour in Wistar rats, exposed to noise stress. Single treatment of kaempferol was more potent than zinc gluconate; but in combination, it acted together with zinc gluconate to produce an improved outcome.

## Supporting information

S1 Table Effect of kaempferol and zinc on open-field parameters in Wistar rats exposed to noise stress on day 1 (Mean ± SEM, n = 6). (DOCX)

S2 Table Effect of kaempferol and zinc on open-field parameters in Wistar rats exposed to noise stress on day 8 (Mean ± SEM, n = 6). (DOCX)

S3 Table Effect of kaempferol and zinc on open-field parameters in Wistar rats exposed to noise stress on day 15 (Mean ± SEM, n = 6). (DOCX)

S4 Table Ameliorative effect of kaempferol, zinc and kaempferol + zinc on neuromuscular performance (Inclined plane) of Wistar rats exposed to noise stress (Mean ± SEM, n = 6). (DOCX)

S5 Table Ameliorative effect of kaempferol, zinc and kaempferol + zinc on motor coordination (Beam walk) of Wistar rats exposed to noise stress (Mean ± SEM, n = 6). (DOCX)

S6 Table Ameliorative effect of kaempferol, zinc and kaempferol + zinc on motor strength (Forepaw grip) of Wistar rats exposed to noise stress (Mean ± SEM, n = 6). (DOCX)

S7 Table Ameliorative effect of kaempferol, zinc and kaempferol + zinc on sensorimotor reflex (Excitability score) of Wistar rats exposed to noise stress (Mean ± SEM, n = 6). (DOCX)
