# Supplementary material for: Kaempferol and zinc gluconate mitigate neurobehavioral deficits and oxidative stress induced by noise exposure in Wistar rats
Source: PLoS One. 2020 Jul 21;15(7):e0236251. doi: 10.1371/journal.pone.0236251 (PMC7373279; doi:10.1371/journal.pone.0236251)
Supplement: S1 Table — (DOCX) [file pone.0236251.s001.docx]

##

## S1 Table: Effect of kaempferol and zinc on open-field parameters in Wistar rats exposed to noise stress on day 1 (Mean ± SEM, n = 6)

| **Day 1** |  |  |  |  |  |
| --- | --- | --- | --- | --- | --- |
|  |  |  | **Group** |  |  |
| **Parameters** | **DW** | **DW+N** | **K+N** | **Zn+N** | **K+Zn+N** |
| **Rearing** | 44.40 ± 2.10 | 52.10 ± 0.60 | 32.20 ± 1.00 | 40.10 ± 6.40 | 37.20 ± 1.44 |
| **Stretching** | 5.00 ± 1.00 | 4.10 ± 2.00 | 6.10 ± 1.40 | 4.50 ± 0.50 | 5.00 ± 0.40 |
| **Defecation** | 3.00 ± 2.20 | 3.10 ± 1.00 | 3.5.00 ± 2.00 | 2.90 ± 0.20 | 3.20 ± 0.10 |
| **Urination** | 2.00 ± 0.16 | 2.40 ± 0.20 | 2.20 ± 0.80 | 2.00 ± 0.00 | 2.0 ± 0.60 |
| **Grooming** | 32.80 ± 0.16 | 23.00 ± 0.50 | 20.00 ± 0.40 | 10.00 ± 0.80 | 15.00 ± 0.40 |
| **Locomotion** | 16.00 ± 0.40 | 4.20 ± 0.20 | 8.40 ± 2.00 | 16.80 ± 4.00 | 12.40 ± 0.20 |
