# Supplementary material for: Kaempferol and zinc gluconate mitigate neurobehavioral deficits and oxidative stress induced by noise exposure in Wistar rats
Source: PLoS One. 2020 Jul 21;15(7):e0236251. doi: 10.1371/journal.pone.0236251 (PMC7373279; doi:10.1371/journal.pone.0236251)
Supplement: S2 Table — (DOCX) [file pone.0236251.s002.docx]

## S2 Table: Effect of kaempferol and zinc on open-field parameters in Wistar rats exposed to noise stress on day 8 (Mean ± SEM, n = 6)

|  |  |  | **Group** |  |  |
| --- | --- | --- | --- | --- | --- |
| **Parameters** | **DW** | **DW+N** | **K+N** | **Zn+N** | **K+Zn+N** |
| **Rearing** | 33.10 ± 8.62 | 30.00 ± 0.42 | 37.90 ± 1.48 | 22.20 ± 4.26 | 37.20 ± 1.44 |
| **Stretching** | 4.50 ± 0.70 | 4.80 ± 1.04 | 4.20 ± 0.80 | 4.40 ± 0.52 | 3.90 ± 0.48 |
| **Defecation** | 4.30 ± 2.22 | 3.90 ± 1.46 | 3.30 ± 2.24 | 3.50 ± 2.60 | 3.89 ± 1.20 |
| **Urination** | 2.20 ± 0.74 | 2.80 ± 0.82 | 1.80 ± 0.55 | 2.80 ± 0.63 | 2.24 ± 0.10 |
| **Grooming** | 15.00 ± 0.20 | 20.48 ± 0.82 | 21.10 ± 0.80 | 11.40 ± 0.25 | 28.10 ± 0.80 |
| **Locomotion** | 39.90 ± 2.67 | 43.60 ± 1.47 | 32.10 ± 1.97 | 21.70 ± 2.52 | 42.60 ± 1.80 |
