# Supplementary material for: Kaempferol and zinc gluconate mitigate neurobehavioral deficits and oxidative stress induced by noise exposure in Wistar rats
Source: PLoS One. 2020 Jul 21;15(7):e0236251. doi: 10.1371/journal.pone.0236251 (PMC7373279; doi:10.1371/journal.pone.0236251)
Supplement: S3 Table — (DOCX) [file pone.0236251.s003.docx]

## S3 Table: Effect of kaempferol and zinc on open-field parameters in Wistar rats exposed to noise stress on day 15 (Mean ± SEM, n = 6)

|  |  |  | | |  | |  | |  | |
| --- | --- | --- | --- | --- | --- | --- | --- | --- | --- | --- |
|  |  |  | | | **Group** | |  | |  | |
| **Parameters** | **DW** | | **DW+N** | **K+N** | | **Zn+N** | | **K+Zn+N** | |  |
| **Rearing** | 25.02 ± 4.31 | 27.00 ± 2.21 | | | 29.00 ± 4.25 | | 18.40 ± 2.64 | | 29.20 ± 6.44 | |
| **Stretching** | 3.20 ± 0.70 | 5.10 ± 0.50 | | | 3.80 ± 0.20 | | 4.20 ± 2.40 | | 3.30 ± 3.10 | |
| **Defecation** | 4.00 ± 2.4 | 4.30 ± 2.24 | | | 2.49 ± 0.90 | | 2.00 ± 6.40 | | 2.00 ± 0.00 | |
| **Urination** | 1.20 ± 0.50 | 2.80 ± 0.00 | | | 2.00 ± 0.40 | | 2.20 ± 1.60 | | 2.00 ± 0.20 | |
| **Grooming** | 22.20 ± 4.80 | 30.0 ± 3.20 | | | 25.20 ± 5.20 | | 20.00 ± 2.10 | | 36.50 ± 0.70 | |
| **Locomotion** | 26.20 ± 1.40 | 20.50 ± 0.80 | | | 28.10 ± 1.60 | | 15.20 ± 1.60 | | 28.00 ± 1.40 | |
