# Supplementary material for: Kaempferol and zinc gluconate mitigate neurobehavioral deficits and oxidative stress induced by noise exposure in Wistar rats
Source: PLoS One. 2020 Jul 21;15(7):e0236251. doi: 10.1371/journal.pone.0236251 (PMC7373279; doi:10.1371/journal.pone.0236251)
Supplement: S4 Table — (DOCX) [file pone.0236251.s004.docx]

## S 4 Table: Ameliorative effect of kaempferol, zinc and kaempferol + Zinc on neuromuscular performance (Inclined plane) of Wistar rats exposed to noise stress (Mean ± SEM, n=6)

|  |  |  | **Group** |  |  |
| --- | --- | --- | --- | --- | --- |
| **Day** | **DW** | **DW+N** | **K+N** | **Zn+N** | **K+Zn+N** |
| **1** | 64.17 ± 2.01 | 64.17 ± 1.54 | 65.00 ± 1.83 | 64.17 ± 1.54 | 64.17 ± 1.54 |
| **8** | 64.17 ± 1.54 | 60.83 ± 3.01 | 62.50 ± 2.14 | 60.83 ± 2.39 | 63.33 ± 1.67 |
| **15** | 65.00 ± 1.29 | 58.33 ± 2.11 | 62.50 ± 1.71 | 61.67 ± 2.11 | 64.17 ± 2.01 |
