# Supplementary material for: Kaempferol and zinc gluconate mitigate neurobehavioral deficits and oxidative stress induced by noise exposure in Wistar rats
Source: PLoS One. 2020 Jul 21;15(7):e0236251. doi: 10.1371/journal.pone.0236251 (PMC7373279; doi:10.1371/journal.pone.0236251)
Supplement: S5 Table — (DOCX) [file pone.0236251.s005.docx]

## S 5 Table: Ameliorative effect of kaempferol, zinc and kaempferol + Zinc on motor coordination (Beam walk) of Wistar rats exposed to noise stress (Mean ± SEM, n=6)

|  |  |  | Group |  |  |
| --- | --- | --- | --- | --- | --- |
| **Day** | **DW** | **DW+N** | **K+N** | **Zn+N** | **K+Zn+N** |
| **1** | 4.00±0.94 | 10.83±1.40 | 6.83±1.33 | 9.5±0.76 | 5.33±1.45 |
| **8** | 6.17±1.87 | 11.9±1.18 | 5.67±1.94 | 6.33±1.91 | 4.33±1.41 |
| **15** | 5.00±0.94 | 19.50±0.92 | 5.83±1.33 | 6.60±1.89 | 3.83±1.35 |
