# Supplementary material for: Kaempferol and zinc gluconate mitigate neurobehavioral deficits and oxidative stress induced by noise exposure in Wistar rats
Source: PLoS One. 2020 Jul 21;15(7):e0236251. doi: 10.1371/journal.pone.0236251 (PMC7373279; doi:10.1371/journal.pone.0236251)
Supplement: S6 Table — (DOCX) [file pone.0236251.s006.docx]

##

## S 6 Table: Ameliorative effect of kaempferol, zinc and kaempferol + Zinc on motor strength (Forepaw grip) of Wistar rats exposed to noise stress (Mean ± SEM, n=6)

|  | **Group** | | | |  |  |
| --- | --- | --- | --- | --- | --- | --- |
| **Day** | **DW** | **DW+N** | **K+N** | **Zn+N** | **K+Zn+N** |  |
| **Day 1** | 90.00 ± 2.44 | 85.83 ± 3.05 | 89.67 ± 1.84 | 85.00 ± 2.63 | 89.83 ± 1.45 | |
| **Day 8** | 86.67 ± 3.09 | 70.83 ± 4.19 | 69.67 ± 1.38 | 64.83 ± 1.76 | 78.67 ± 2.55 | |
| **Day 15** | 82.33 ± 0.95 | 51.67 ± 2.29 | 71.11 ± 2.69 | 70.00 ± 1.00 | 78.33 ± 2.69 | |

## S 7 Table: Ameliorative effect of kaempferol, zinc and kaempferol + Zinc on sensorimotor reflex (Excitability score) of Wistar rats exposed to noise stress (Mean ± SEM, n=6)

|  |  | **Group** | | |  |
| --- | --- | --- | --- | --- | --- |
| **Day** | **DW** | **DW+N** | **K+N** | **Zn+N** | **K+Zn+N** |
| **Day 1** | 4.83 ± 0.17 | 4.83 ± 0.17 | 4.83 ± 0.17 | 4.83 ± 0.17 | 4.83 ± 0.17 |
| **Day 8** | 4.53 ± 0.17 | 4.87 ± 0.32 | 4.78 ± 0.34 | 4.50 ± 0.22 | 4.23 ± 0.17 |
| **Day 15** | 4.44 ± 0.17 | 4.98 ± 0.40 | 4.67 ± 0.21 | 4.63 ± 0.21 | 3.93 ± 0.01 |
