# Supplementary material for: Kaempferol and zinc gluconate mitigate neurobehavioral deficits and oxidative stress induced by noise exposure in Wistar rats
Source: PLoS One. 2020 Jul 21;15(7):e0236251. doi: 10.1371/journal.pone.0236251 (PMC7373279; doi:10.1371/journal.pone.0236251)
Supplement: S7 Table — (DOCX) [file pone.0236251.s007.docx]

##

## S 7 Table: Ameliorative effect of kaempferol, zinc and kaempferol + Zinc on sensorimotor reflex (Excitability score) of Wistar rats exposed to noise stress (Mean ± SEM, n=6)

|  |  | **Group** | | |  |
| --- | --- | --- | --- | --- | --- |
| **Day** | **DW** | **DW+N** | **K+N** | **Zn+N** | **K+Zn+N** |
| **Day 1** | 4.83 ± 0.17 | 4.83 ± 0.17 | 4.83 ± 0.17 | 4.83 ± 0.17 | 4.83 ± 0.17 |
| **Day 8** | 4.53 ± 0.17 | 4.87 ± 0.32 | 4.78 ± 0.34 | 4.50 ± 0.22 | 4.23 ± 0.17 |
| **Day 15** | 4.44 ± 0.17 | 4.98 ± 0.40 | 4.67 ± 0.21 | 4.63 ± 0.21 | 3.93 ± 0.01 |
